# Bayesian Estimation of Potential Performance Improvement Elicited by Robot-Guided Training

**DOI:** 10.3389/fnins.2021.704402

**Published:** 2021-10-21

**Authors:** Asuka Takai, Giuseppe Lisi, Tomoyuki Noda, Tatsuya Teramae, Hiroshi Imamizu, Jun Morimoto

**Affiliations:** ^1^Department of Brain Robot Interface, Computational Neuroscience Laboratories, Advanced Telecommunications Research Institute International (ATR), Kyoto, Japan; ^2^Mechanical and Physical Engineering Course, Graduate School of Engineering, Osaka City University, Osaka, Japan; ^3^Department of Psychology, The University of Tokyo, Tokyo, Japan; ^4^Department of Cognitive Neuroscience, Brain Information Communication Research Laboratory Group, ATR, Kyoto, Japan; ^5^Graduate School of Informatics, Kyoto University, Kyoto, Japan

**Keywords:** haptic guidance, skill level, motor training, robotic teaching, human-robot interaction

## Abstract

Improving human motor performance via physical guidance by an assist robot device is a major field of interest of the society in many different contexts, such as rehabilitation and sports training. In this study, we propose a Bayesian estimation method to predict whether motor performance of a user can be improved or not by the robot guidance from the user’s initial skill level. We designed a robot-guided motor training procedure in which subjects were asked to generate a desired circular hand movement. We then evaluated the tracking error between the desired and actual subject’s hand movement. Results showed that we were able to predict whether a novel user can reduce the tracking error after the robot-guided training from the user’s initial movement performance by checking whether the initial error was larger than a certain threshold, where the threshold was derived by using the proposed Bayesian estimation method. Our proposed approach can potentially help users to decide if they should try a robot-guided training or not without conducting the time-consuming robot-guided movement training.

## Introduction

Collaboration between robots and humans can expand human capabilities and has been investigated on the applicability in fields ranging from rehabilitation to collaborative manufacturing. Many different approaches have been developed to train human movements with robots by providing motor instructions and feedback. For this kind of application, it is essential to predict whether an individual responds to a specific robotic training (Sigrist et al., 2013) before actual training to avoid wasted time and effort, but such estimation methods have not been established.

Furthermore, the efficacy of robotic instruction through haptic sense has not been sufficiently investigated while the haptic interface that provides motor instructions to human users has been long-term explored (Mussa-Ivaldi et al., 1985; Sigrist et al., 2013). The effect of somatosensory feedback has been compared to that of visual guidance. For example, Feygin et al. examined haptic guidance in short-term training to learn novel three-dimensional (3D) circular trajectories. They found that haptic training alone was less effective than lone visual training for positional reproduction performance (Feygin et al., 2002). Liu et al. also studied the short-term performance of tracking novel 3D circular trajectories. They found that haptic input in addition to visual demonstration did not improve the tracing error compared to the visual-alone condition (Liu et al., 2006). Wong et al. examined skill learning in 3-day consecutive haptic interface training of drawing two-dimensional (2D) trajectories. They rather found that additional haptic demonstration showed greater improvements than visual-alone conditions (Wong et al., 2012).

On the other hand, previous studies suggested that haptic instructions seem to be beneficial to initially less-skilled participants (Sigrist et al., 2013). Marchal-Crespo et al. (2010) found that initially less-skilled participants significantly improved their steering skills after training using the haptic guided driving task. However, the previous studies did not provide a systematic approach either to verifying the grouping depending on individual initial skill level or selecting a specific boundary to estimate potential motor improvement. They rather found a linear correlation between the initial skill level and its change after robotic haptic interaction (Marchal-Crespo et al., 2010, 2017; Duarte and Reinkensmeyer, 2015). Although only Duarte and Reinkensmeyer used information criteria and identified the relevance of initial skills to the change, they have not tried to define the boundary value.

Identifying the boundary promises positive training effects for target users of each task or the type of robotic training. This study proposes an identification method to evaluate the dependence of the training effect on the initial skill level by modeling the skill level change between before and after receiving the haptic guidance training. We verify the grouping’s validity based on model fitness and propose a systematic method to set a theoretically sound boundary value.

## Materials and Methods

### Bayesian Modeling of the Skill Level Change

To provide the boundary for estimating whether motor performance of a user can be improved or not, we first verify the skill level change model differs between individuals depending on their initial skill level. For this, we referred to Sigrist’s summary. Sigrist et al. (2013) suggested that position haptic guidance may be useful for novices or less skilled. This can be interpreted as the skill level change model that allows to vary both the intercept and slope by the initial participant’s skill. We prepared four different hypothetical models, as shown in Table 1. To model changes in skill level for an evaluation metric, we employed the Bayesian statistical modeling based on Markov Chain Monte Carlo (MCMC) with a No-U-turn sampler and variational inference (Salvatier et al., 2016). Specifically, the linear models in Table 1 have both the intercept (α) and slope (β), which were allowed to vary between models. The analysis used the following basic formula:

(1) $${\hat{y}}_{i\,j}=\alpha_{i\,j\,\left[k\right]}+\beta_{i\,j\,\left[k\right]}\,x_{k}+\epsilon_{k}$$

**TABLE 1:** Models of the skill level change.

Model number (i)	Formula	Description
1. Pooled	${\hat{y}}_{1\,j}=\alpha_{1}+\beta_{1}\,x_{i}$	• Independent to initial skill • Effective equally to everybody
2. Varying slope	${\hat{y}}_{2\,j}=\alpha_{2}+\beta_{2\,j}\,x_{i}$	• Independent to initial skill • Effective differently for individual
3. Varying intercept	${\hat{y}}_{3\,j}=\alpha_{3\,j}+\beta_{3}\,x_{i}$	• Dependent on the initial skill • Effective equally to everybody
4. Varying intercept and slope	${\hat{y}}_{4\,j}=\alpha_{4\,j}+\beta_{4\,j}\,x_{i}$	• Dependent on the initial skill • Effective differently for individual

where *i* is the number of the model, *j* is the index of each participant, *k* is the index of each trial, $\hat{y}$ is the variable of interest, and *x* is the session variable (that is, 0: first, 1: second session). The formula for a participant (*j*) is illustrated in Figure 1A.

**FIGURE 1:**
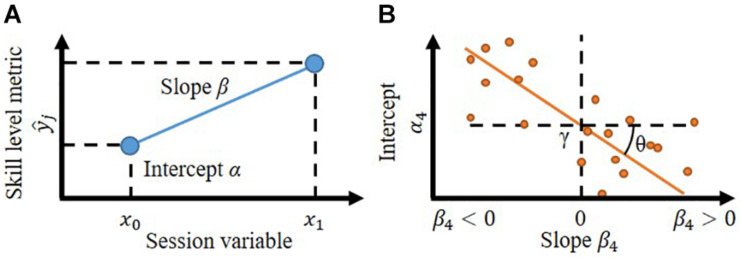
**(A)** Linear modeling of the skill level change of the participant (*j*). The skill level metric ($\hat{y}$) here in this study is the error between the target movement and performed movement. The intercept (α) is the initial skill level (*x_0_*). The slope (β) represents the change in skill level. **(B)** The linear relationship between the intercept and slope. Each dot represents a linear model of each participant. The linear relationship allows to set the boundary (γ) to divide participants into two groups systematically; those whose skill level improves (β < 0) or those who decline (β > 0).

Model 1 (in Table 1) has the participant independent intercept and slope, which means the change in metric is independent of the participants and their initial skill level. If this is the case, all participants can attain the benefit of robotic instruction. This means all participants have the same skill level change model and highly likely the lowest model fitness among the four. Model 2 has a participant-dependent slope, which means the change in skill varies among participants but cannot be predicted by their initial skill level. Model 3 has the participant-dependent intercept, which means that the robotic instruction can equally affect their skill change regardless of their initial skill level. Although Model 3 is ideal as an instruction, it is highly unlikely to have a high fitness to the haptic instruction. Model 4 has varying intercepts and slopes. Thus, the skill level change can be predicted by their initial skill level. If the metric’s fitness to Model 4 is greater than the others, it signifies that the haptic instruction is beneficial to initially less skilled participants. This supports the initial skill-based grouping statistically. Spontaneously, it also suggests that the initial performance can result in motor improvements after receiving instructions from the existing dataset.

### Linear Relationship Between the Intercept and Slope to Define the Boundary

If the skill level change model differs between individuals, we can derive the boundary using the relationship between the initial metric (that is, the intercept) and the change in the metric (that is, the slope). Hence, we included the following linear equation in model 4:

(2) $$\alpha_{j}=\theta \,\beta_{j}+\gamma$$

The formula is illustrated in Figure 1B. A non-zero θ would highlight a significant relationship between α and β, while a non-zero γ would signify that for some participants, performance improved (β < 0), while for other participants, performance declined (β > 0). Thus, γ is the boundary of the initial skill level. γ was estimated simultaneously while estimating α and β by MCMC, so posthoc analysis was not needed.

The complete probabilistic model is defined as follows:

(3) $$Y∼𝒩\,\left(\mu_{i},\sigma^{2}\right)\;\mu_{i}∼\alpha_{\mathrm{ij}}+\beta_{\text{i}\,j}\cdot X\;\sigma ∼\left|𝒞\,\left(5\right)\right|$$

(4) $$\alpha_{i\,j}∼\{\begin{array}{cc} 𝒩\,\left(0,10^{-5}\,\right) & i=1,2\\ 𝒩\,\left(\mu_{\alpha \,i},\sigma_{\alpha }^{2}\,\right) & i=3,4 \end{array}\,\mu_{\alpha \,i}∼\{\begin{array}{cc} 𝒩\,\left(0,10^{-5}\,\right) & i=3\\ \theta \cdot \beta_{i\,j}+\gamma & i=4 \end{array}\,\sigma_{\alpha }∼\left|𝒞\,\left(5\right)\right|$$

(5) $$\sigma_{\theta }∼ℱ\;\sigma_{\gamma }∼ℱ$$

(6) $$\beta_{\text{i}\,j}∼\left\{ \begin{array}{cc} 𝒩\,\left(0,10^{-5}\,\right) & i=1,3\\ 𝒩\,\left(\mu_{\beta },\sigma_{\beta }^{2}\,\right) & i=2,4 \end{array}\right.\;\mu_{\beta }∼𝒩\,\left(0,10^{-5}\,\right)\;\sigma_{\beta }∼\left|𝒞\,\left(5\right)\right|$$

where all the quantities defined in the previous paragraph still hold, *Y* represents the outcomes (skill level metric), *X* represents the predictors (that is, 0: first, 1: second session), 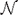 is the Gaussian distribution, |
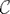
(5)| is a Half-Cauchy distribution with parameter 5, and ℱ is an uninformative (flat) prior. All the parameters of the prior distributions were based on the default settings of the probabilistic modeling software (Salvatier et al., 2016).

### Sample Dataset: Experiment With a Haptic Interface

The above model was applied to the experimental data of participants who interacted with a robot-assisted motor training system from our laboratory, which guided the participant’s hand to show the procedure to process an actual motor task of interest.

#### Participants

Participants included 20 healthy right-handed adults (17 men, 3 women; age range: 21–34 years; mean ± standard deviation [SD] = 24.017 ± 2.596). The handedness was determined by a verbal inquiry based on the Edinburgh inventory. All participants provided written informed consent before participation. The ATR Review Board Ethics Committee approved the study protocol.

#### Task and Apparatus

**FIGURE 2:**
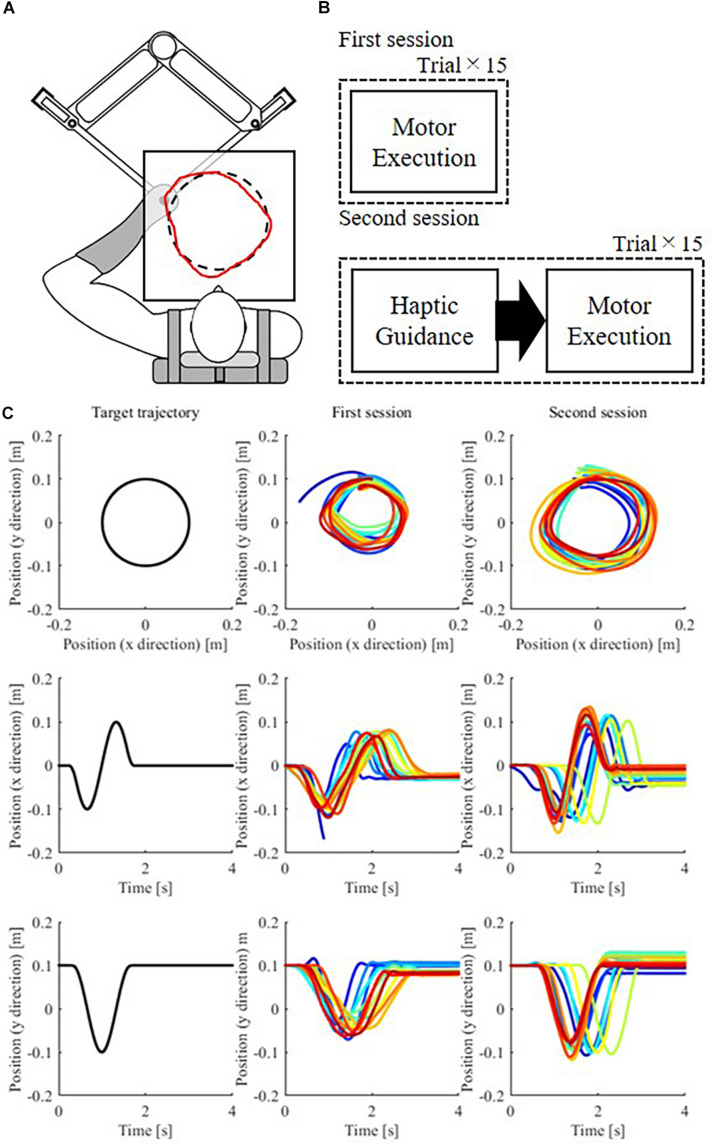
**(A)** Motor task and apparatus. Participants were asked to draw a true circle (dashed line) using their left hand within 2 s. A manipulandum located under the table provided haptic guidance. The participants could hold a handle on the manipulandum, and it moved to guide their hand in the desired direction. The red line shows a representative example of a handwritten trajectory. Both the target circle and drawn figures were hidden from the participants during motor execution, such that the participants never saw the actual hand position. **(B)** Procedure. The participants completed 15 trials in which they drew a circle with score feedback at the end of each trial. Next, the participants completed 15 trials in which they first received haptic guidance from the robot, that is, allowed the robot to move their hand in the desired trajectory, and then executed the drawing movement by themselves without being assisted by the robot. Finally, they received their score at the end of each trial. **(C)** Target movement and executed movements by a participant at both sessions in the *x–y* plane and its time trajectory in the *x* and *y* directions. Early trials are plotted as blue traces, and subsequent trials are denoted by “warmer” colors.

The target task involved drawing a true circle of 10 cm radius on a horizontal plane using one’s left hand. We selected our task referencing existing studies with healthy subjects introduced in section “Introduction,” especially Wong et al. (2012). Feygin et al. (2002) identified an interference between the visual and haptic modals, so we decided not to provide visual feedback to our participants during haptic feedback. The subject’s hand is hidden under a white table, on top of which additional information can be visualized using a projector. Participants were asked to complete the drawing within approximately 2 s. They started drawing the circle from the 12 o’clock position and moved in a counter-clockwise direction. All the task details were consistent with those in our previous experiment (Takai et al., 2018). A robotic manipulandum located under a white table guided the target movement (Figure 2A). The table prevented the participant from viewing their hand as it moved. The robot was programmed to provide negligible resistance to movement while the participants were drawing. For safety, the robot stopped moving when the force applied at the end effector exceeded the prescribed range or when the handle left a specified safe area.

#### Haptic Feedback

The manipulandum moved the participant’s left hand along the targeted movement trajectory. Participants received proprioceptive afferent information during the entire movement. The robot handle moved at a constant velocity outside the acceleration/deceleration (A/D) period, set to 0.2 s after it starts and before it finishes the movement. The target circle was visible during movement guidance. As with our previous study (Takai et al., 2018), the participants could not see their hand’s current position or the robot’s end-effector at any moment. During the robotic guidance, the participants were instructed not to move their arms with or against the robot’s movement. However, the participants were not completely passive to the guidance, as they maintained the posture of their arms to avoid coming in contact with the table.

#### Score Feedback

We evaluated the drawn circles by the participants and fed back the score to the participants soon after each trial. The equations used to calculate the score are as follows:

(7) $$\text{ERR}\,\left(t\right)=\sqrt{{\left(x_{\text{hand}}\,\left(t\right)-x_{\text{target}}\,\left(t\right)\right)}^{2}+{\left(y_{\text{hand}}\,\left(t\right)-y_{\text{target}}\,\left(t\right)\right)}^{2}}$$

(8) $$\text{Score}\,\left(t\right)=100\,\frac{E_{\max.}-\text{ERR}\,\left(t\right)}{E_{\max.}}$$

(9) $$\text{Trial}\,\text{Score}=\frac{1}{t_{e}-t_{s}}\,\sum_{t=t_{s}}^{t_{e}}\text{Score}\,\left(t\right)$$

where ERR(*t*) is the error between the hand and target position at time *t*, *x*_hand_(*t*) and *y*_hand_(*t*) are the coordinates of the hand position at time *t*, and *x*_target_(*t*), *y*_target_(*t*) are the coordinates of the target position at time *t*, *t_s_* is the starting time, and *t_e_* is the ending time. *E*_*max.*_ is the maximum allowed error and is set to be the same as the target circle radius.

#### Experimental Design

At the beginning of the experiment, participants were familiarized with the task by observing a human instructor performing the task. Participants have been told the diameter of the target circle is 10 cm. Although we did not explicitly show ideal velocity profiles to a subject, we asked the subject to generate the hand movement with a constant speed and also informed that the task duration was 2 s.

Subsequently, they underwent the experimental procedure as shown in Figure 2B. During the first session, the participants were instructed to reproduce the target movements in terms of both position and velocity as accurately as possible without any assistance from the manipulandum. The participants’ active movements were measured for 15 trials (Figure 2B, top). Before starting a trial, the target circle is projected on the table for approximately 3 s. Subsequently, the circle is removed, and no visual information about the circle size, speed, or the current hand position is provided. We evaluated the circles drawn by the participants in each trial. The average error between the target and the performed movement was normalized such that the values ranged from 0 to 100 (as shown in Eq. (8)). After each trial, the score was projected on the table for approximately 3 s using a projector. Subjects are asked to improve their score. While the target circle and the current hand position are also visualized with the score, the performed trajectory was not shown to the participants.

In the second session, participants received haptic guidance from the manipulandum. Subjects are instructed to memorize the position and velocity of the guided motion as accurately as possible in preparation for the following motor execution. Figure 2B bottom shows that each trial consisted of one haptic guided presentation by the robot and one participant’s motor execution. There were 5 s intervals before and after the haptic guidance. The score was shown to the participant at the end of each trial, similar to the first session. This session continued until the participants completed 15 trials (Figure 2B, bottom).

Both session trials in which the movement exceeded the specified safe area were not evaluated. However, they were counted to reach a predetermined number of 15 trials. The average number of trials for evaluation was 14.8 (SD 0.44) in the first session and 14.1 (SD 1.47) in the second session.

### Skill Level of Each Trial

Skill level was evaluated as the positional distance from the target circle as well as the difference between the performed velocity and the actual target velocity. Previous studies (Feygin et al., 2002; Liu et al., 2006; Lüttgen and Heuer, 2012; Wong et al., 2012) suggested that tracking performances of different physical variables such as position and velocity in a trajectory learning task could be sensitive to different types of modalities such as vision and haptics, respectively. These studies identified that the shape accuracy improved more in visual training, while haptic training was better for training the temporal aspects. Since our robot-guided training provides haptic feedback to a user, tracking performances of velocity profiles would be improved more than that of position trajectories. Thus, we separately evaluated position and velocity tracking performances to investigate the effectiveness of the robot-guided haptic feedback. For each trial, the position and velocity errors were evaluated for 1.46 s, starting at the moment when the participants’ hand left the start zone, within a circle with a diameter of 3 cm centered at 12 o’clock position. The position and velocity errors defined in Eqs (10, 11) were only used for analysis. Note that executed movements only by participants among all trials in the second session were evaluated.

(10) $$E_{p}=\frac{1}{t_{e}-t_{s}}\,\sum_{t=t_{s}}^{t_{e}}\left|r_{h}\,\left(t\right)-r\right|$$

(11) $$E_{v}=\frac{1}{t_{e}-t_{s}}\,\sum_{t=t_{s}}^{t_{e}}||{\text{v}}_{h}\,\left(t\right)-\text{v}\,\left(t\right)||$$

where *E_p_* is the positional error from the target, *t_s_* is the starting time, *t_e_* is the ending time, *r*_*h*_(*t*) is the current hand radius with respect to the workspace center at time *t*, and *r* = 10 cm is the constant target radius. *E_v_* is the velocity error from the target velocity ||**v**|| = 37.62 cm/s. **v**_*h*_(*t*) is the current hand velocity with respect to the workspace center at time *t*.

## Results

### Evaluation of Models’ Fitness to the Sample Dataset

The experimental result of a representative participant is shown in Figure 2C. The participant drew the circle smaller than the target in the first session, but the size increased after receiving the haptic guidance in the second session. The participants (*n* = 20) mean errors as a function of the trial numbers decreases within each session, except the position error in the first session (Supplementary Figure 1). After the first session, still 4.3 mm error remained as the lowest position error. Therefore, the lowest position-error participant could further improve the tracking performance. In other words, the remained error indicated that the obtained results were not due to a ceiling effect on the performance. Meanwhile, there were marginally positive relationships between the mean of 15 trials among each participant’s position and velocity errors in both sessions (Supplementary Figure 2).

**FIGURE 3:**
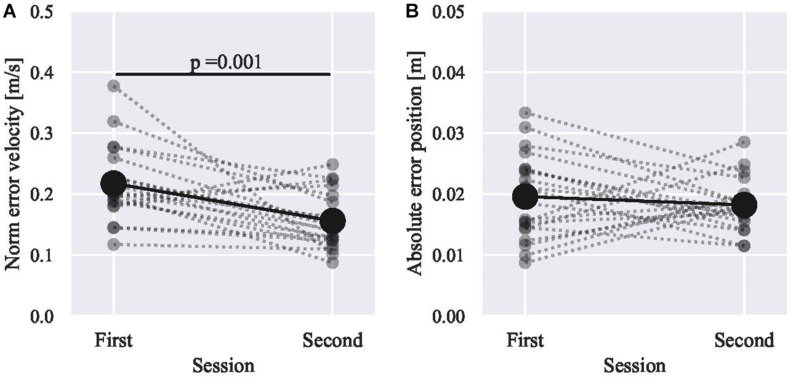
The skill level change between sessions. The average of all participants metric is shown in black, and that of each participant is superimposed in gray. The metric is **(A)** the norm of error velocity and **(B)** the absolute error in position. The target peripheral speed is 0.376 m/s, and the radius of the target circle is 10 cm. The paired two-sample tests are Student’s *t* for the velocity and Wilcoxon signed-rank test for the position.

The position and velocity errors are shown in Figure 3. Looking into the change in skill level for each participant (gray lines in Figure 3), the slope ranges from strong positive to strong negative. The lack of significant improvement in positional accuracy could have been due to the use of average data for all participants instead of classifying participants into groups. We fitted the models in Table 1 to the metric to determine whether such grouping is reasonable. The results are shown in Table 2 for velocity and Table 3 for position.

**TABLE 2:** Models’ fitness of norm of error velocity.

Models	WAIC (Mean ± SD)
1. Pooled	−1,129 ± 16
2. Varying slope	−1,175 ± 17
3. Varying intercept	−1,217 ± 18
4. Varying intercept and slope	−1,311 ± 12

**TABLE 3:** Models’ fitness of absolute positional error.

Models	WAIC (Mean ± SD)
1. Pooled	−3,733 ± 22
2. Varying slope	−3,774 ± 22
3. Varying intercept	−3,856 ± 17
4. Varying intercept and slope	−4,035 ± 16

The models’ fitnesses were evaluated using the widely applicable information criterion (WAIC; Watanabe, 2010). The smaller the WAIC, the better the fit. By the leave-one-subject-out (LOSO) analysis, both criteria were tested 20 times, and the mean and SD are as shown in Tables 2, 3. Model 4 with varying intercepts and slopes had the best fit for both velocity and position metrics. Therefore, it was fair to divide participants based on their initial skill level.

### Deriving the Boundary

**FIGURE 4:**
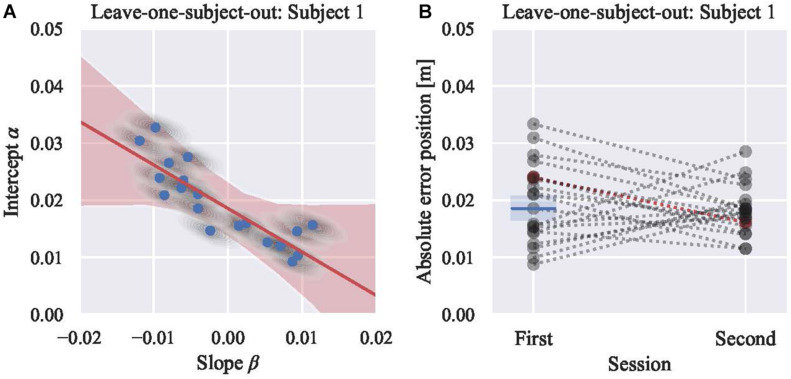
**(A)** Posterior predictive plot of model 4 using 19 participants’ absolute position errors (leaving subject 1). Each gray shaded area represents multiple samples from the posterior [the intercepts (α) and the slopes (β)] of each subject, and each blue circle shows the average of each area. Red shaded area represents regression lines for all samples, and red line shows their average. The boundary (γ, the intercept of the red line) is 0.019 m with the credible interval (94%) from 0.017 to 0.021 m. **(B)** Visualization of classifying results. Subject 1, showing in red marker, is tested. Rest of 19 participants are showing in gray markers. Blue line shows the boundary derived from model 4 in **(A)**. Blue shaded area shows the 94% credible interval. Mean absolute position error of Subject 1 in the first session was above the boundary. Thus, subject 1 is classified into a group that is expected to improve the performance in the second session. Actual mean absolute position error in the second session is less than that of the first session.

**FIGURE 5:**
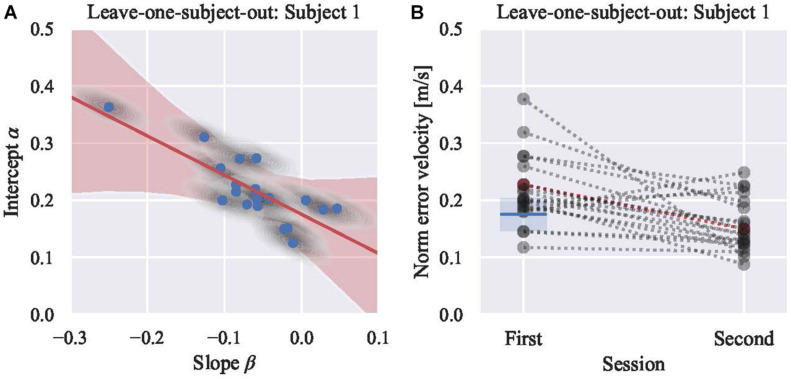
**(A)** Posterior predictive plot of model 4 using 19 participants’ norm of error velocity (leaving subject 1). Each gray shaded area represents multiple samples from the posterior [the intercepts (α) and the slopes (β)] of each subject, and each blue circle shows the average of each area. Red shaded area represents regression lines for all samples, and red line shows their average. The boundary (γ, the intercept of the red line) is 0.175 m/s with the credible interval (94%) from 0.147 to 0.204 m/s. **(B)** Visualization of classifying results. Subject 1, showing in red marker, is tested. Rest of 19 participants are showing in gray markers. Blue line shows the boundary derived from model 4 in **(A)**. Blue shaded area shows the 94% credible interval. Mean norm of error velocity of Subject 1 in the first session was above the boundary. Thus, subject 1 is classified into a group that is expected to improve the performance in the second session. Actual mean norm of error velocity in the second session is less than that of the first session.

**FIGURE 6:**
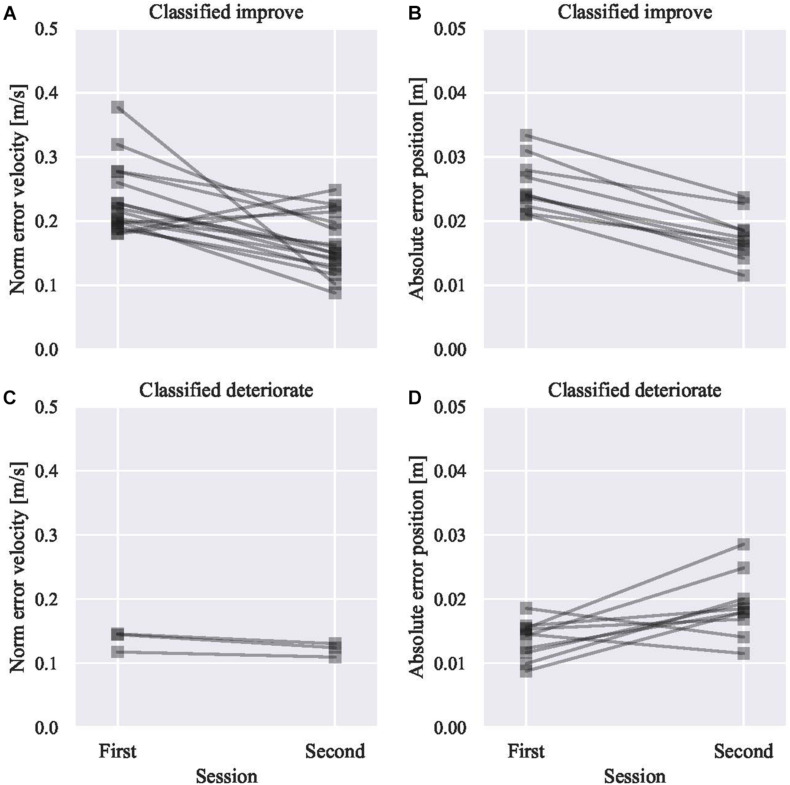
**(A)** The classified subject for improving velocity error based on LOSO method. Subjects were above the boundary in the first session, so they are predicted to improve their performance in the second session. Most of them scored fewer errors in the second session. **(B)** Subject predicted as to improve positional error. **(C)** Subject predicted as to deteriorate velocity error. **(D)** Subject predicted as to deteriorate positional error.

Subsequently, we inspected the linear model between the intercept (α) and slope (β) to derive the boundary (γ). The LOSO analysis was conducted, and the sample result excluding subject 1 is as shown in Figures 4, 5 (Figure 4 for the position and Figure 5 for the velocity). After fitting the linear model, the distributions of θ and γ do not include zero. Thus, a significant relationship between α and β was identified, and it signified that for some participants, performance improved (β < 0), while for other participants, performance declined (β > 0). As shown in Figure 4B, the slope (β) of subjects who have an initial error above the boundary γ are negative; however, those with an initial error below are positive. Based on the confusion matrix, the accuracy of classification was 0.9 for the position and 0.7 for the velocity models. The F measure was 0.91 for the position model and 0.82 for the velocity model. The excluded subject’s performance in the second session was predicted by the initial skill level. As shown in Figure 6, the subjects are well classified into two groups based on the boundary.

### Group-Based Haptic Guidance Effect

**FIGURE 7:**
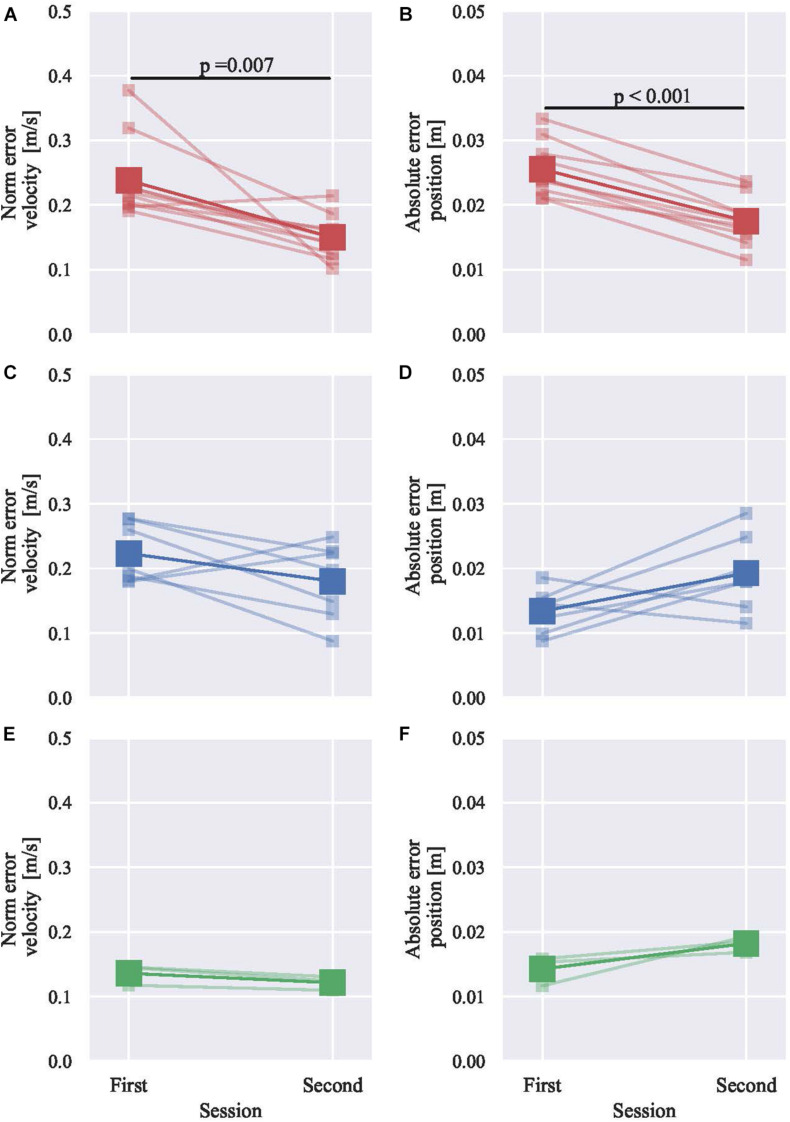
The skill level change between sessions with participants grouping based on the derived boundaries. **(A)** Red group velocity. **(B)** Red group position. **(C,D)** Blue group and **(E,F)** Green group. The paired two-sample tests are Wilcoxon signed-rank test in **(A)** and Student’s *t*-test in **(B)**.

The 20 participants were allocated into three groups based on position and velocity boundary. The numbers of participants in each group are shown in Table 4. Figure 7 shows the skill level change between sessions of all the three groups. The participants in the red group were initially low-skilled in terms of both position and velocity, while the participants in the green group were initially high-skilled. The blue group was initially low-skilled in terms of velocity but was highly skilled in terms of position. The initially low-skilled participants in terms of position but highly skilled in terms of velocity were not found in the dataset.

**TABLE 4:** Classification in groups.

	Below position threshold	Above position threshold
Above velocity threshold	Number of subjects = 7 (Color in Figure 7: Blue)	Number of subjects = 10 (Color in Figure 7: Red)
Below velocity threshold	Number of subjects = 0	Number of subjects = 3 (Color in Figure 7: Green)

Without grouping, the efficacy of haptic guidance was not significant, especially in positional accuracy, as shown in Figure 3B. However, by grouping, the red group showed significant improvements in both metrics (the velocity and position). On the contrary, the green group shows a minor deterioration in terms of position, while a minor improvement in terms of velocity was also observed. These results suggest that the initially low-skilled participants significantly improved their skill level. Regarding the blue group, the initially low-skilled aspect (velocity) improved; however, the initially high-skilled aspect (position) did not improve, while both did not significantly change. Those of who increased the velocity error also increased the positional error (2 out of 3 subjects).

## Discussion

Due to the increasing demand to improve motor performance via human-robot collaboration, numerous different approaches have emerged; however, not all of them guarantee motor performance improvements (Williams and Carnahan, 2014). It would be useful and efficient if the chance of success for a user could be estimated prior to training. Our study proposes a versatile method that can statistically elaborate on the relationship between performance improvements and the person’s initial skill level.

### Identifying Target People Through the Statistical Grouping Method

In this study, we have proposed a Bayesian estimation method for examining different linear models that explain the relationship between the initial skill level and its change. By comparing these models, the most appropriate model to explain this relationship can be identified. This provides a non-heuristic but hypothesis-based approach to analyze the benefit of interest. Moreover, hypothetical models, that is, the relationship between motor performance and the initial skill level, can be explicitly implemented and even compared to identify which model the data with maximum likelihood.

Four different models have been examined in this study (Table 1). These are fully against (Model 1), partially against (Models 2 and 3), or in agreement with Sigrist’s summary (Model 4). If the metric’s fitness to Model 4 is greater than the others, it can signify that the skill level change model differs between individuals. Hence, the performance improvement is a function of the initial skill level and statistically supports the initial skill-based grouping. We used WAIC for model evaluation, which aims to select a model that makes good predictions, rather than the likelihood ratio test, which aims for the safe rejection of the null hypothesis and cannot show that the alternative hypothesis is good (Posada and Buckley, 2004). As a result, WAIC is the lowest in Model 4 with varying intercepts and slopes than the other models for both velocity and position metrics. Therefore, it statistically supports dividing participants based on their initial skill level. The skill level metric ($\hat{y}$) used in this study is the error between the target and performed movement. Thus, the method is neither parameter- nor task-dependent and is expected to work in a wide range of applications.

Grouping of participants either qualitatively or quantitatively has been explored in previous studies. For example, to define participants’ experiences, authors generally used classification terms, such as Novice and Expert (Beilock et al., 2002). While in another study, the motor skill level is sometimes referred to as participants’ symptoms, for example in autism, where the patients have motor difficulties to some extent, or typically developed (Staples and Reid, 2010). In other studies, participants who scored on a motor test under a specified threshold (Marchal-Crespo et al., 2010) or the median among the participants (Etnier and Landers, 1998) are grouped as less-skilled. Participants are also sometimes grouped based on quantiles (Malina et al., 2007). Grouping into an equal number of participants (Yamamoto et al., 2019) has also been introduced insofar. However, the reason why grouping is reasonable is not well explained. Metric-based approaches have also been introduced (Hook et al., 2004; Gruber et al., 2006; Dose et al., 2007). These studies identified unique and best metrics among many options to identify handwriting. They developed feature-based classification algorithms. However, the method to verify clustering relies on subjective labeling. Aharonson and Krebs (2012) used the no-labeling method but still had to run an exhaustive search. Thus, heuristic-based approaches could not be avoided in previous studies. Limitations regarding our approach are discussed in section “Challenges and Prospects on Model Interpretation Regarding Potential Motor Improvements.”

### Defining the Skill Level Boundary Through Linear Modeling of Its Change

We included a linear relationship between the coefficients of the linear model (Figure 1B) to derive the boundary. By inferring the parameters using the Bayesian inference, non-zero coefficients provided evidence of a linear relationship. This shows an effective boundary to identify those that can benefit from haptic guidance. The parameters are inferred in consideration of the uncertainty under the limited data assuming the existence of a certain true value for each parameter because the Bayesian approach takes into account the uncertainties of parameter values while providing exact inference. In contrast, most maximum likelihood (or least squares) estimation fixes the parameter values though there is considerable uncertainty (Punt and Hilborn, 2001). The boundary that is suitable for practical use needs to be estimated from a small number of data samples -as is the case in exploratory experiments with human subjects (Sabatini and Mannini, 2016; Kim et al., 2017)- and to be robust for new data, and it is better not to vary with each re-estimation. Bayesian estimates obtained from MCMC procedures are appropriate in small samples (Dunson, 2001; Gray et al., 2015). Since Bayesian models accommodate unobserved variables (in our case, gamma) with associated uncertainty (Dunson, 2001), we can confidently build a threshold.

The linear relationship between the intercept (α) and the slope (β) fits the skill-level metric change of absolute error in position as well as the norm of error velocity. As a result, the boundary (γ) is derived with sufficiently low WAIC. Non-zero θ clearly shows that for some participants’ performance improved, while for others, performance declined. The difference in metric change trends between the first and second session is also visible between the participants who are above and below the boundary (Figures 4B, 5B). Such Bayesian estimation using a complex model cannot be done with simple linear regression (Dunson, 2001; Punt and Hilborn, 2001). Although the metrics relationship may fit more with a non-linear model or may need more data (Figures 4A, 5A), these results prove the concept of model-based interpretation of the motor training effects and potential. In future studies, an extended (for example, mixed effect, order effect) model-based inference could be applied.

In the scenario of using the estimated parameters in this study, an examiner of the haptic guided training can classify subjects with confidence because the boundary is provided with the credible interval as the most likely value from the computed posterior distribution. When a subject’s initial skill is at the vicinity of the boundary, the posterior probability distribution (the certainty of the boundary) can support the examiner’s judgment. The estimated boundary value fixed with considerable uncertainty (Punt and Hilborn, 2001) has little merit in the above interpretation. Hespanhol et al. (2019) demonstrated that the credible interval is more natural and easy-to-interpret than the frequentist intervals. Even in a small sample size, the percent of the credible interval that contained the true population mean is higher than that of the confidence interval (Gray et al., 2015).

Previous studies have already identified the linear correlation of initial skill level to its change following robotic haptic interaction (Marchal-Crespo et al., 2010, 2017; Duarte and Reinkensmeyer, 2015). Although only Duarte and Reinkensmeyer (Duarte and Reinkensmeyer, 2015) performed information criteria and identified the relevance of initial skills to changes other than fixed effects, the statistical test does not answer the use of the identified effect in real-world applications. Looking at rehabilitation studies, many studies have been made regression models for predicting trial-by-trial change in impairment (Casadio and Sanguineti, 2012) or long-term effect, including daily-life usage-dependent changes implicitly (Reinkensmeyer et al., 2016). Although the potential benefit of making a prognosis based on the clinical scores and the brain images, these studies do not predict whether a patient responds to a specific intervention or a robotic treatment. Meanwhile, Schweighofer and colleagues not only statistically identified potential predictor of changes in clinical score after arm rehabilitation but also derived a functional threshold for who can benefit (Schweighofer et al., 2009). They successfully proved their concept, but the accuracy was not as high as in this study. This highlights the importance of verification using different hypothetical models rather than examining a single model.

A linear relationship between initial skill level and changes after robotic haptic interaction may be found in various tasks, ranging from driving a car (Marchal-Crespo et al., 2010), golf patting (Duarte and Reinkensmeyer, 2015), leg rehabilitation (Marchal-Crespo et al., 2017), and tasks related to upper arm motor functionality, as are, in this study. Therefore, the linear modeling method may be applicable and useful in other motor tasks and training approaches.

### Efficacy of Haptic Guidance in Motor Training

In previous studies, haptic training methods were evaluated based on the means of all participants’ metrics (Feygin et al., 2002; Liu et al., 2006; Lüttgen and Heuer, 2012; Wong et al., 2012). Without grouping, as shown in Figure 3, the norm of error velocity decreased (improved) after haptic guidance training. However, the absolute error in position shows no change on average. Therefore, the training effect suggested from our dataset without grouping is questionable as is in line with previous studies. For example, haptic training improved the timing aspect (Feygin et al., 2002; Lüttgen and Heuer, 2012) with short-term training but not for positional error (Wong et al., 2012). These consistencies prove that the dataset is not peculiar or an artificial one prepared to explain the proposed method.

This study verified the fairness in dividing participants based on the initial skill level using a derived boundary. By grouping, initially low-skilled participants significantly improved their average skill level regarding both position and timing aspects. The training’s effectiveness and identified target participants are consistent with a previous study that used the heuristics grouping method (Marchal-Crespo et al., 2010). Haptic guidance is a major approach in robotic rehabilitation to facilitate motor functional recovery (Marchal-Crespo and Reinkensmeyer, 2009; Sigrist et al., 2013). This may be an appropriate approach for patients who have lost motor skills.

For high-skilled participants, their performance did not change much. This is consistent with previous studies; for example, “*Benefit of guidance-based training was not detected for the more skilled young/old participants*” (Marchal-Crespo et al., 2010). Some previous studies explained this by referring to the challenge point theory (Guadagnoli and Lee, 2004). The theory states that task difficulty should be appropriately adjusted to meet the participant’s skill level to maximize the training effect. However, this study explains this differently using the derived boundary and explains that performance deterioration may result from difficulty in recognizing the difference between a goal and their movements. High-skilled participants make a very small error from the goal movement but need to identify the error only through somatosensory information. The error is in the same range of the correctly identifiable difference between the reference and test, as reported by Wilson et al. (2010). Since information is successfully processed only when uncertainty is reduced (Fitts, 1954), unreliable haptic guidance for them may not result in motor improvements. Meanwhile, high-skilled participants may improve their performance using score feedback that is specific to the feature to be enhanced or using alternative haptic interaction approaches, for example, error amplification (Milot et al., 2010; Duarte and Reinkensmeyer, 2015; Marchal-Crespo et al., 2017).

The motor performance of the participants in the blue group was partially improved by the haptic guidance. This is consistent with previous experiments that showed learning of timing (Marchal-Crespo et al., 2010), rather than spatial. Participants might be trapped with the speed-accuracy trade-off as the difference in speed to be a difference in the difficulty level of the task (Shmuelof et al., 2012). In other words, the positional accuracy deteriorated because of improved speed accuracy. In this study, the participants can obtain better scores if they attempt to reduce position error at the cost of velocity error or vice versa because the score accounts for both positional and velocity performance. One possible solution might be to feedback velocity and position score separately.

### Challenges and Prospects on Model Interpretation Regarding Potential Motor Improvements

In this study, we have applied the modeling method to sample data of 20 participants and interpreted the outcome to divide participants into discrete groups. Grouping analysis provided a detailed interpretation of the efficacy of haptic guidance for each participant at the specific initial skill level, as discussed in section “Efficacy of Haptic Guidance in Motor Training.” For other tasks, all subjects may improve skill level similarly (that is, no boundary exists). This would make the fit of Model 4 worse or equal to the others. Besides, this method may help to find other kinds of structures in larger data. When the fit of Model 4 is better than the others, there are two possible phenomena: the participant-dependent training effect and the regression toward the mean. Both can be expressed by Model 4; however, they are separable, as the former has a large mean slope in the absolute and the latter has a small one. Nonetheless, this approach would be valuable for exploring the data.

To fit the Bayesian linear model, it requires datasets *a priori*, similar to other data-driven methods. Also, the boundary is highly dependent and influenced by the task. These limitations are common to the studies presented previously; for example, Gruber et al. (2012) made a handedness classifier. Nevertheless, it is beneficial for trainees as they can perceive the possible outcome before continuing the ineffective and time-consuming training. There is, for example, a possible solution to alternate robot approaches to fit the individuals at any level to guarantee the motor improvements (Brown et al., 2016), but our solution is to help identify responders who can benefit from existing approaches. The interpretation can also be useful in assigning suitable next motor skill training protocols, not only for neuro-rehabilitation (Aharonson and Krebs, 2012) but also for skill development manufacturing (Ma, 2014), for establishing personalized and comprehensive motor training programs.

## Conclusion

In this study, we proposed a Bayesian estimation method for examining models that describe the changes in the skill level of haptic guidance training and deriving a boundary for dividing participants into initial skill-level groups. Results showed that we were able to predict whether a novel user can improve the performance by checking that the user’s initial skill level was larger than the boundary. We have also demonstrated that the general idea/heuristic suggested by previous studies can be systematically evaluated. Such methods may be essential to select an effective approach for individuals among other different approaches.

## Data Availability Statement

The datasets generated for this study are available on request to the corresponding author.

## Ethics Statement

The studies involving human participants were reviewed and approved by The ATR Review Board Ethics Committee. The patients/participants provided their written informed consent to participate in this study.

## Author Contributions

AT, TN, HI, and JM contributed to the study design and project supervision. AT, GL, TN, and JM participated in the experimental design. AT, TN, and TT performed data acquisition. AT and GL performed data analysis, interpretation of results, and prepared the manuscript. All authors have read and approved the final manuscript.

## Conflict of Interest

The authors declare that the research was conducted in the absence of any commercial or financial relationships that could be construed as a potential conflict of interest.

## Publisher’s Note

All claims expressed in this article are solely those of the authors and do not necessarily represent those of their affiliated organizations, or those of the publisher, the editors and the reviewers. Any product that may be evaluated in this article, or claim that may be made by its manufacturer, is not guaranteed or endorsed by the publisher.
