# Supplementary material for: Bayesian Estimation of Potential Performance Improvement Elicited by Robot-Guided Training
Source: Front Neurosci. 2021 Oct 21;15:704402. doi: 10.3389/fnins.2021.704402 (PMC8567031; doi:10.3389/fnins.2021.704402)
Supplement: Supplementary file 1 [file Image_1.pdf]

Supplementary document

Figures and captions

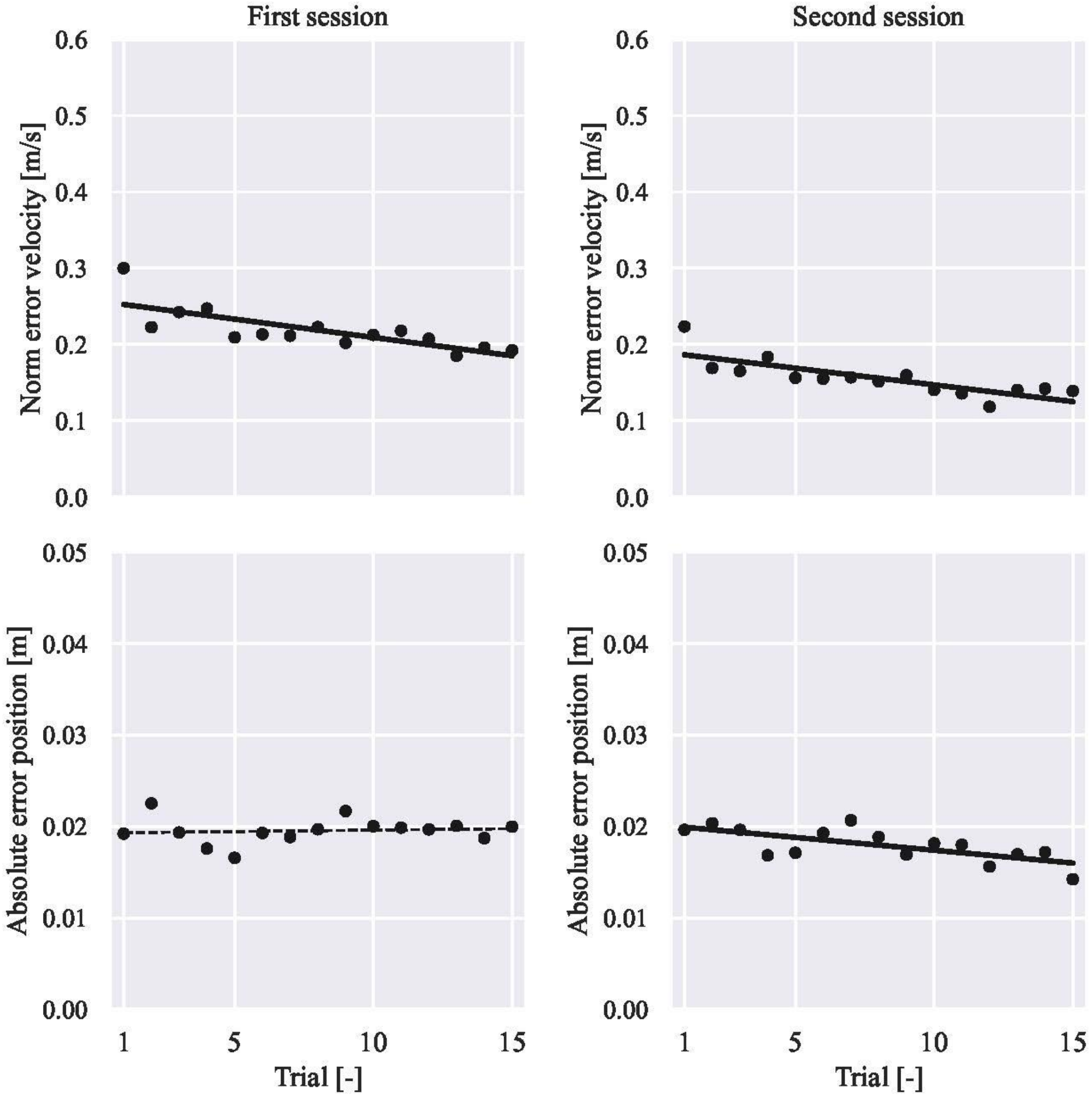

Supplements figure 1: Participants (n = 20) mean errors as a function of trial numbers. Black markers and lines are drawn by using all participants' data in each session. Dashed line shows that the linear regression of black markers was not significant, and solid line shows that the regression was significant ($p < 0.05$).

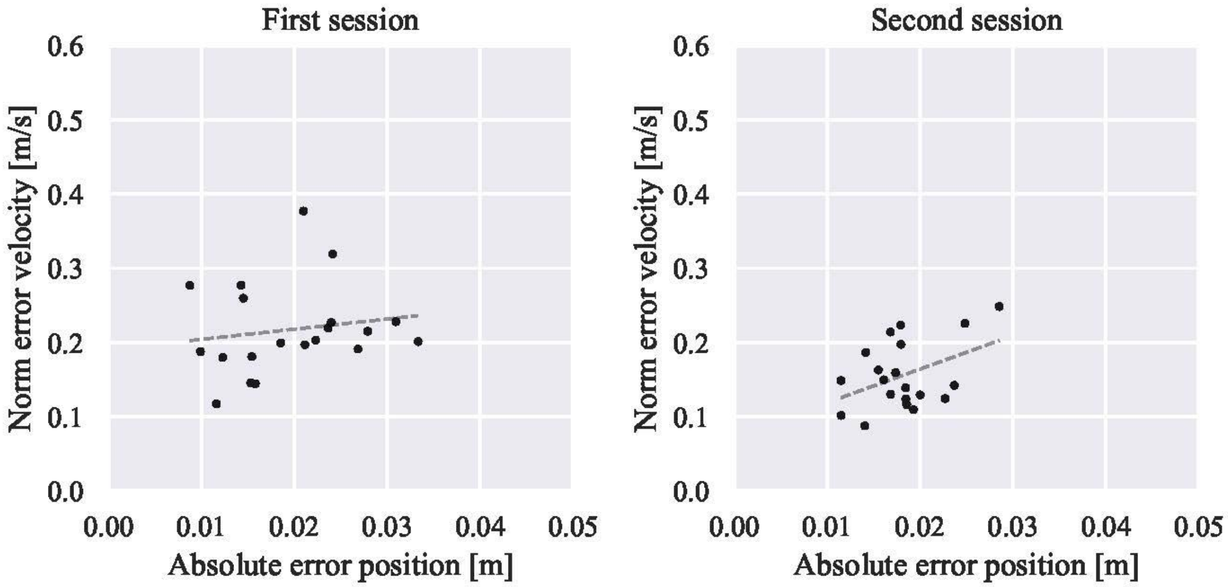

Supplements figure 2: Relationship between participant's position and velocity error. Left panel shows the error relationships in the first session, and right panel shows that in the second session. Each marker represents the mean error of 15 trials of each participant. Linear regression was not significant ($p > 0.05$) in both sessions.
